# Neutralizing antibodies explain the poor clinical response to Interferon beta in a small proportion of patients with Multiple Sclerosis: a retrospective study

**DOI:** 10.1186/1471-2377-9-54

**Published:** 2009-10-13

**Authors:** Emilia Sbardella, Valentina Tomassini, Claudio Gasperini, Francesca Bellomi, Luca Ausili Cefaro, Vincenzo Brescia Morra, Guido Antonelli, Carlo Pozzilli

**Affiliations:** 1 Departments of Neurological Sciences, "Sapienza" University, Rome, Italy; 2 FMRIB Centre, The University of Oxford, Department of Clinical Neurology, John Radcliffe Hospital, Oxford, UK; 3 Department of Neurology, San Camillo Hospital, Rome, Italy; 4 Experimental Medicine and Pathology, Section of Virology, "Sapienza" University, Rome, Italy; 5 Department of Neurological Sciences, "Federico II" University, Naples, Italy

## Abstract

**Background:**

Neutralizing antibodies (NAbs) against Interferon beta (IFNβ) are reported to be associated with poor clinical response to therapy in multiple sclerosis (MS) patients. We aimed to quantify the contribution of NAbs to the sub-optimal response of IFNβ treatment.

**Methods:**

We studied the prevalence of NAbs in MS patients grouped according to their clinical response to IFNβ during the treatment period. Patients were classified as: group A, developing ≥ 1 relapse after the first 6 months of therapy; group B, exhibiting confirmed disability progression after the first 6 months of therapy, with or without superimposed relapses; group C, presenting a stable disease course during therapy. A cytopathic effect assay tested the presence of NAbs in a cohort of ambulatory MS patients treated with one of the available IFNβ formulations for at least one year. NAbs positivity was defined as NAbs titre ≥ 20 TRU.

**Results:**

Seventeen patients (12.1%) were NAbs positive. NAbs positivity correlated with poorer clinical response (*p *< 0.04). As expected, the prevalence of NAbs was significantly lower in Group C (2.1%) than in Group A (17.0%) and Group B (17.0%). However, in the groups of patients with a poor clinical response (A, B), NAbs positivity was found only in a small proportion of patients.

**Conclusion:**

The majority of patients with poor clinical response are NAbs negative suggesting that NAbs explains only partially the sub-optimal response to IFNβ.

## Background

The clinical efficacy of Interferon beta (IFNβ) therapy in Multiple Sclerosis (MS) has been demonstrated consistently in large, randomized, placebo-controlled trials [1]. However, a proportion of treated patients ranging from 7% to 49% show a poor clinical response [2].

Mechanisms underlying IFNβ activity in MS are only partially known. There is evidence suggesting that the interaction between IFNβ and its receptor is responsible for beneficial effects of IFNβ [3,4]. Exposure to IFNβ, however, can result in the development of antibodies against the IFNβ [5,6]. Neutralizing antibodies (NAbs) interact with the biologically active sites of the IFNβ molecule, preventing the interaction of IFNβ with its receptor and downstream effects. Binding antibodies (BAbs) may bind to several different antigenic epitopes of the IFNβ molecule, some of which are not involved in the activation of IFNβ receptors [7]. Moderate or high levels of BAbs developed during the first year of IFNβ treatment may predict of the subsequent development of NAbs [8].

The development of NAbs can reduce or abrogate IFNβ bioavailability, as revealed by decreased levels of biological markers of IFNβ bioactivity, such as neopterin, β_2_-microglobulin, and myxovirus resistance protein A, and the reduction in IFNβ bioavailability may depend on NAbs titres [9-13]. In treated MS patients IFNβ bioactivity decreases as NAbs develop, returning to the normal levels when NAbs levels fall [14]. In NAbs-negative patients an *in vivo *biological response to IFNβ is present, which is not detectable in patients with high NAbs titres [15]. Although the relationship between induction of biological markers and IFNβ clinical activity is not known, this evidence suggests that NAbs detected by validated *in vitro *assays may have consequences *in vivo*.

NAbs against IFNβ have been largely studied as one of the factors responsible for poor clinical response to therapy in MS. Although many studies have shown that NAbs reduce IFNβ efficacy, as measured by MRI and clinical disease activity [5,10,16-25], the clinical relevance of NAbs, especially at low titres, in MS patients treated with IFNβ remains debatable [26,27].

It remains, however, to be established whether NAbs play a major role in determining a poor clinical response to IFNβ. Here, we aim to quantify the percentage of NAbs positivity among sub-optimal responder patients to determine to what extent NAbs contribute to IFNβ response in MS. This could inform clinicians on the contribution of NAbs detection to IFNβ response and support clinical decision making.

## Methods

### Patients

We included patients with a diagnosis of relapsing-remitting MS (RRMS) or secondary-progressive MS (SPMS) [28] receiving IFNβ treatment as a first line therapy and undergoing routine assessment at the MS University Outpatient Services of Rome, "Sapienza" University, and Naples, "Federico II" between January 2004 and December 2005.

Patients aged from 18 to 55 years were eligible if they had a score of 6.0 or lower on the Expanded Disability Status Scale (EDSS) [29]. Patients with RRMS should have experience at least two relapses supported by history and confirmed by physical examination, during the 2 years prior to the commencement of therapy. Patients with SPMS were eligible if they had experienced a sustained disability progression for at least 6 months after an initial RRMS course of disease. Only patients taking IFNβ therapy for at least 12 months prior to study entry were included. RRMS patients were undergoing treatment with standard doses of one of the IFNβ formulations available; treatment for SPMS was restricted to IFNβ-1b, the only drug licensed in Italy for treatment of SPMS. The Ethics Committees of the two University Hospitals approved the study.

### Demographic and clinical assessments

Demographic and clinical data were regularly recorded at each 6-month visit on the on-site database.

A *relapse *was defined as the appearance or re-appearance of one or more symptoms attributable to MS, accompanied by objective deterioration lasting at least 24 hours on neurological examination, in the absence of fever, and preceded by neurological stability for at least 30 days [30]. The relapse were assessed by a trained neurologist and in the event of a disabling relapse, patients received a standard course of steroids (methylprednisolone, 1 g/day intravenously) for 5 days.

### Definitions of response to IFNβ therapy

We defined three groups of patients according to the clinical response to IFNβ, based on disability progression and the number of relapses over the study period:

#### Group A

patients experiencing relapses without confirmed disease progression during the treatment period before NAbs testing. Relapses occurred in the first 6 months of therapy were not counted.

#### Group B

patients who experienced an increase of ≥ 1.0 point of the EDSS, or ≥ 0.5 points if baseline EDSS score was ≥ 5, with confirmed disease progression at 6 months, persisting for at least 2 consecutive scheduled visits, with or without superimposed relapses during the treatment period; the EDSS at the time of the relapse was not considered in the definition of disease progression. Patients without a confirmatory exam were excluded from the study.

#### Group C

patients with a clinically stable disease, i.e. without relapses and/or disability progression during the study period. Within each group, patients were stratified according to the type of IFNβ formulation with which they were treated.

Twelve out of 141 patients (8.5%) were treated with IFNβ-1a 30 mcg (Avonex^®^; Biogen Idec, Cambridge, MA), administered intramuscularly once a week (hereafter 'Avonex'); 36 (25.5%) were treated with IFNβ-1b 8 MIU (Betaferon^®^; Bayer-Schering Pharma, Berlin, Germany), administered subcutaneously every other day (hereafter 'Betaferon'); 48 (34.0%) were treated with IFNβ-1a 22 mcg (Rebif 22^®^; Merck Serono International S.A., Geneva, Switzerland, an affiliate of Merck KGaA, Darmstadt, Germany), administered three times a week (hereafter 'Rebif 22'); and 45 (31.9%) patients were treated with IFNβ-1a 44 mcg (Rebif 44^®^; Merck Serono International S.A., Geneva, Switzerland, an affiliate of Merck KGaA, Darmstadt, Germany), administered three times a week (hereafter 'Rebif 44').

### NAbs assessment

**Figure 1:**
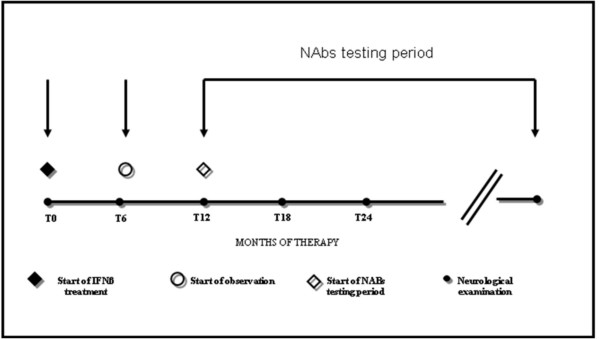
**Timeline of the study**. The blood sample to test for the presence of NAbs was taken once during the study, at least 12 months after starting IFNβ treatment. Relapses or EDSS progression occurred in the first 6 months of therapy were not counted.

A blood sample was taken once during the study, at least 12 months after starting IFNβ treatment, to test for the presence of NAbs (Figure 1). Patients did not receive corticosteroids in the month preceding blood sampling and had not been treated with immunosuppressive drugs associated with IFNβ. At the time of blood sampling, EDSS was assessed. Response to therapy, i.e. patients' classification into groups A, B or C, was defined on the basis of the neurological assessment performed at the time of blood sampling for the NAbs test.

Blood samples were allowed to clot at room temperature for an hour prior to centrifugation. Sera were collected and stored at -20°C in small aliquots until analysis. The presence of NAbs to IFNβ was tested using a cytopathic effect (CPE) assay against 10 IU of recombinant IFNβ-1a or IFNβ-1b [31]. Sera were inactivated at 56°C for 30 minutes before titration. Two-fold serial dilutions (starting from 1:10) of sample or control sera in 60-μL volumes were incubated with 60 μL of 20 IU/mL of each type of IFNβ at 37°C for an hour. Next, 100 μL of individual sample mixtures were added to duplicate monolayers of human lung carcinoma (A549) cells in 96-well microtitre plates. After 18 to 24 hours of culture and extensive washing, cells were exposed to encephalomyocarditis virus, followed by incubation at 37°C for 24 hours. Controls included a titration of the IFN preparation used in the assay.

Antiviral activity and its neutralization were assessed on the basis of virus-induced CPE. To quantify the CPE, cells were stained with crystal violet in 20% ethanol, followed by elution of dye taken up by the cells with 33% acetic acid. The extent of virus-induced CPE, its inhibition by recombinant IFNβ, and the reversal of this by NAbs were shown by the amount of dye eluted from each sample. Titres were calculated using Kawade's method [32], and expressed as ten-fold reduction units (TRU), namely, the dilution of serum that reduces 10 laboratory units (LU) per mL of IFN to 1 LU/mL [33]. Serum samples underwent routine assay and were found to be free of endogenous or residual IFN activity. None of the sera showed an intrinsic antiviral activity and therefore no sera were excluded.

Patients were classified according to their NAbs status into NAbs-negative and NAb-positive patients. NAbs-positivity was defined as a titre of ≥ 1:20 neutralizing units. Once classified as NAbs-positive or NAbs-negative, patients kept their classification for the duration of the study according to the definition "anytime positive, always positive" [16].

### Statistical analysis

The influence of NAbs on clinical efficacy, including clinically documented relapses and confirmed disability progression, was tested. Differences between groups were analyzed using nonparametric tests (Pearson chi-squared test) for qualitative and categorical variables, and parametric tests (Student's *t-*test for independent samples or univariate analysis of variance) for quantitative variables. The Bonferroni *post-hoc *test was used for multiple comparisons and the paired *t*-test was used to compare the same variables at different times. Analyses were performed using SPSS version 8.0 software (SPSS Inc., Chicago, USA). Significance was set at *p *< 0.05.

## Results

### Baseline demographic and clinical characteristics

Data were collected retrospectively from 141 patients (90 women and 51 men) with a mean (± standard deviation, SD) age of 38.6 (± 9.7) years, disease duration since the first clinical event suggestive of MS, of 10.98 (± 6) years and a median EDSS score of 2 (range: 0-6.0). The mean (± standard deviation, SD) of treatment duration before Nabs testing was 4.3 (± 2.1) years (range 1-10).

Patients treated with Betaferon were older and had higher baseline EDSS scores than patients treated with Rebif 22 or Rebif 44 (Table 1). Durations of disease and treatment were also longer in the Betaferon- than in Rebif-treated patients.

**Table 1:** Baseline characteristics of the patients grouped according to the IFNβ formulation

**Characteristics**	**Avonex** **n = 12**	**Rebif 22** **n = 48**	**Rebif 44** **n = 45**	**Betaferon** **n = 36**	***p***
Age (years)	39.9 ± 10.8 (27-64)	38.7 ± 9.1 (23-58)	35 ± 8.4 (21-58)	42.6 ± 10.1 (25-67)	0.04

Disease duration (years)	12.3 ± 6.6 (5-24)	9.9 ± 5.3 (2-24)	9.7 ± 5.4 (3-28)	13.6 ± 6.8 (3-34)	0.01

Therapy duration (years)	4.7 ± 1.7 (2-8)	4.2 ± 1.6 (1-9)	3.2 ± 1.8 (1-10)	5.7 ± 2.5 (1-10)	0.001

EDSS score	1.8 ± 1.0 (1-4.5)	1.9 ± 1.0 (0-5)	2.0 ± 1.2 (0-5.5)	2.65 ± 1.5 (0-6)	0.02

All values are mean ± SD (range)

When the entire cohort of patients was grouped according to the clinical response, within each group (n = 47), 4 patients were treated with Avonex, 12 with Betaferon, 16 with Rebif 22, and 15 with Rebif 44. The three response groups were similar in terms of baseline demographic and clinical characteristics (Table 2).

**Table 2:** Baseline characteristics of the patients grouped according to the clinical response to IFNβ

**Characteristics**	**Group A** **(n = 47)**	**Group B** **(n = 47)**	**Group C** **(n = 47)**	***p***
Age (years)	38.2 ± 10 (23-64)	40.4 ± 10.8 (21-67)	37.3 ± 7.9 (23-56)	0.29

Disease duration (years)	10.6 ± 5.3 (3-24)	12.5 ± 6.3 (4-34)	9.9 ± 6.3 (2-28)	0.11

Therapy duration (years)	4.4 ± 2.3 (1-10)	4.1 ± 1.9 (1-10)	4.3 ± 2.2 (1-10)	0.79

All values are means ± SD(range)

*Group A: *patients experiencing at least one relapse after the first 6 months of therapy without confirmed disease progression. *Group B: *patients experiencing a sustained disability progression (increase of ≥ 1.0 point on the EDSS, or ≥ 0.5 points if baseline EDSS score was ≥ 5.5) with or without superimposed relapses during the treatment period. *Group C: *patients with a stable disease, i.e., no relapse and/or disability progression.

### NAbs status and clinical response to IFNβ therapy

The presence of NAbs was tested in all patients after starting IFNβ treatment (median 4 years, range 1-10 years). Fifty-nine (42%) patients were tested between the first and the third year after the beginning of IFNβ, while 82 (58%) were tested after the third year of treatment.

### Whole cohort

NAbs were detected in 17 patients (12.1%). Eight out of 17 NABs-positive patients were tested after the third year of therapy. Seventy-four out of 124 NAbs-negative (59.7%) patients were tested after the third year of therapy.

Baseline demographic and clinical characteristics did not differ between NAbs-positive and NAbs-negative patients (Table 3), with the exception of age, as NAbs-negative patients were younger (*p *< 0.05).

**Table 3:** Baseline characteristics of the patients grouped according to the presence of Nabs

**Characteristics**	**NAbs status**		***P***
		
	**Negative** **(n = 124)**	**Positive** **(n = 17)**	
Gender			
Female	80 [64.5]	10 [58.8]	0.65
Male	44 [35.5]	7 [41.2]	0.65

Age (years)	38 ± 9.4 (21-64)	43 ± 10.8 (31-67)	0.046

Disease duration (years)	11 ± 5.8 (2-29)	11 ± 7.4 (3-34)	0.92

Treatment duration (in years)	4 ± 2.2 (1-10)	4 ± 1.7 (1-7)	0.14

IFNβ type			0.96
Avonex (n = 12)	11 [8.9]	1 [5.9]	
Betaferon (n = 36)	31 [25.0]	5 [29.4]	
Rebif 22 (n = 48)	42 [33.8]	6 [35.3]	
Rebif 44 (n = 45)	40 [32.3]	5 [29.4]	

Data are percentage [%] or mean ± SD (range)

### Treatment group

When NAb-positivity was assessed on the basis of the treatment group, one patient (8.3%) treated with Avonex, 5 (13.9%) treated with Betaferon, 6 (12.5%) treated with Rebif 22, and 5 (11.1%) treated with Rebif 44 were found to be NAbs-positive (Table 3). No significant differences were observed between NAbs-positive and NAbs-negative patients according to IFNβ preparation.

### Response group

**Figure 2:**
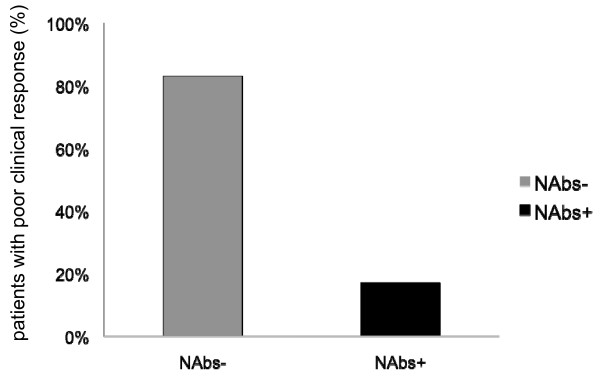
**Prevalence of NAbs according to the clinical response**. Within the groups of patients showing poor clinical response (Group A and B), only 16 out of 94 patients (17.0%) are NAb-positive and 78 (83%) are NAb-negative. This suggests that NAbs presence explains only partially the poor clinical response to IFNβ.

When the presence of NAbs was considered according to the clinical response to therapy, a significant interaction was found between clinical response and NAbs development (*p *< 0.04). The prevalence of NAbs was higher in groups showing poor clinical response (Group A: 17.0%; Group B: 17.0%) than in patients with clinically stable disease (Group C: 2.1%). However, within groups with a suboptimal response (Group A and Group B) only 16 out of 94 (17%) patients were NAbs-positive, whereas 78 were NAbs-negative (Figure 2). Forty-nine out of 78 (63%) NAbs-negative patients underwent the blood sampling after the third year of therapy.

## Discussion

This study shows that the prevalence of NAbs is higher in patients who experience relapses or disability progression during IFNβ therapy than in patients with stable disease. However, NAbs seem to explain only partially the poor clinical response to IFNβ treatment, as only a small proportion of patients with poor response to IFNβ become Nabs-positive while on disease modifying treatment.

The development of NAbs has been reported to reduce treatment efficacy [5,16,34] and their clinical relevance is correlated with titre and persistence of antibodies. High-titre, persistent NAbs have been consistently shown to impair the activity of IFNβ, whereas low-titre NAbs appear to have a milder effect [26,35-37]. In the present study, NAbs status accounted for a small percentage of non-responders: only 17.0% (16/94) of patients with either relapses or disease progression over the treatment period were NAbs-positive at the time of blood sample. Although the impact of IFNβ is different in RRMS and SPMS [1] and IFNβ mainly acts as anti-inflammatory drug, in our study the prevalence of NAbs was similar in Group A and B. This may suggests that the development of NAbs abrogating IFNβ bioavailability have the same negative influence on disease worsening both in terms of relapses and EDSS progression.

The low prevalence of NAbs in patients with poor response to therapy brings into question the clinical impact of these antibodies on treatment efficacy. Absence of NAbs does not guarantee IFNβ efficacy, as a large proportion of patients who experience relapses or disability progression do not develop NAbs. Indeed, risk factors for a poor response to therapy that are independent from NAbs status have been described. Genetic polymorphisms may constitute intrinsic determinants of individual differences in response to IFNβ [38,39].

The contribution of NAbs to clinical response to IFNβ depends on the definition of responders. Previously, different definitions of non-responders to IFNβ therapy have been proposed but none of them have been validated in long-term follow-up studies [2,40]. A reduction in the number of relapses was used to define responders to therapy. However, this definition is weakened by the phenomenon of 'regression to the mean' [2]. In this study, responders were defined as patients who had never experienced a relapse during treatment. We used disability progression confirmed at 6 months to identify patients with a poor response; this definition has been shown to have good sensitivity and specificity [2].

NAbs development was not predictable on the basis of baseline characteristics. We did not find significant relationships between NAbs status and baseline characteristics. However, younger patients were marginally more likely to remain NAbs-negative than older patients [18].

The overall prevalence of NAbs observed in this study (12.1%) was lower than reported previously [5,15-17], but was similar to that shown in patients treated with IFNβ for 3 to 10 years [41]. Several factors may explain these differences in prevalence.

NAbs appearance may have been influenced by the definition of NAbs positivity. Here we used an "any-time positive, always positive" definition, testing patients only once to determine the presence of NAbs. As we used the single-sample method and obtained samples at variable time intervals from the beginning of therapy ranging from 1 to 10 years, fluctuations in NAbs titres (and NAbs status) cannot be completely ruled out. Previous studies have shown that NAbs can be transitory and that some NAbs-positive patients, particularly those with low titers, may sero-revert to a negative status over time despite ongoing IFNβ treatment [7,16,31]. The low prevalence of NAbs (12.1%) in this study, especially in patients treated with INFβ-1b, may be related to the longer duration of treatment. Indeed, 58 patients were tested for NAbs after the third year of treatment, allowing for sufficient time for NAbs to disappear. Therefore, many of the NAbs-negative patients could have been positive during the first years of treatment. It has been shown that, returning to a NAbs-negative state, treated patients may regain a biologic response to IFNβ [42], but whether this will result in a restoration of the therapeutic efficacy remains unclear. A recent study suggests that after reverting from a NAbs-positive to a NAbs-negative status, the therapeutic effect of IFN-β-1b on relapses is restored [43].

## Conclusion

Although evidence suggests that the development of NAbs can adversely affect IFNβ efficacy, we suggest that NAbs-positivity explains the poor response to IFNβ treatment only in a small proportion of patients with MS and their appearance should be weighted against other factors hampering the response to treatment.

## Competing interests

CP received honoraria for consultancy or speaking from Biogen, Bayer Schering, Merck Serono and research grants from Merck Serono; GA received research grants from Merck Serono, Biogen and Bayer Schering; VBM received research grants from Merck Serono, and Bayer Schering; CG received honoraria for consultancy or speaking from Biogen, Bayer Schering, Meck Serono and research grants from Bayer Schering.

## Authors' contributions

ES, LAC, CP: have made substantial contributions to conception and design, acquisition of data, analysis and interpretation of data. FB: carried out the immunoassays. VT, CG, GA: have been involved in revising the manuscript critically for important intellectual content. All authors have given final approval of the version to be published.

## Pre-publication history

The pre-publication history for this paper can be accessed here:


